# Elevated PDE4C level serves as a candidate diagnostic biomarker and correlates with poor survival in thyroid carcinoma

**DOI:** 10.1038/s41598-024-57533-w

**Published:** 2024-03-21

**Authors:** Ying Wang, Yongsheng Zhang, Yanyan Li, Jing Huang

**Affiliations:** 1 Department of Laboratory Medicine, The First Hospital of Jilin University, Changchun, 130021 China; 2 Center for Reproductive Medicine and Center for Prenatal Diagnosis, The First Hospital of Jilin University, Changchun, 130021 China

**Keywords:** Phosphodiesterase 4, Thyroid carcinoma, Cyclic-adenosine monophosphate, Biomarker, Prognosis, Thyroid cancer, Prognostic markers

## Abstract

Thyroid carcinoma (THCA) is the most common endocrine cancer. Phosphodiesterase (PDE) 4 enzyme family, as specific regulator of cyclic adenosine monophosphate, may play a important role in THCA. However, few studies on PDE4 enzyme family in THCA have been reported yet. Therefore, this study aimed to systematically analyze the changes of PDE4 enzyme family in THCA, and look for potential target for THCA therapy. We systematically analyzed the expression differences, prognostic value, genetic alteration, methylation modification, and the correlation with tumor immune microenvironment of PDE4 family in THCA using several public databases, including TCGA, GEO, GSCA, TNMplot, cBioPortal, DiseaseMeth and TIMER. Besides, functional enrichment analysis and protein–protein interaction (PPI) network of PDE4 family was investigated using Metascape and STRING databases. The expression levels of PDE4A, PDE4B and PDE4D were down-regulated in THCA patients at different cancer stages, while the expression level of PDE4C was significantly up-regulated. Moreover, THCA patients with higher PDE4C expression had shorter progress free survival compared with those with lower PDE4C expression. The low genomic alteration frequencies and mildly increased methylation levels of PDE4 family were found in THCA patients. Except for PDE4A, the expression levels of PDE4B, PDE4C and PDE4D could affect many immune cells infiltration during THCA progression. Four PDE4 subtypes were all enriched in cAMP catabolic process. Nevertheless, PDE4C was not enriched in the cAMP binding signal pathway, and PDE4B was not enriched in the G alphas signaling events. Notably, PDE4C participated in cAMP metabolic process by regulating adenylate cyclases (ADCYs), which involved ADCY1, ADCY5, ADCY6, ADCY8 and ADCY9. The findings of this study provide a partial basis for the role of PDE4 family in the occurrence and development of THCA. In addition, this study also suggested that PDE4C might be a potential prognostic marker of THCA, which could serve as a reference for future basic and clinical research.

## Introduction

Thyroid carcinoma (THCA) is the most common endocrine cancer and the fastest growing cancer worldwide^1,2^. Most THCA are well-differentiated carcinomas, which are slow to progress and have an excellent prognosis, but up to 30% of patients experience recurrence^3^. THCA patients may potentially benefit from the utility of diagnosis markers in more extensive initial surgery to include central compartment lymph node dissection to prevent tumor recurrence^4^. Therefore, the discovery of new markers could provide more personalized treatments for THCA patients and help to improve the understanding of related molecular pathogenesis^5,6^.

Phosphodiesterases (PDEs) are a family of 11 enzyme families responsible for the hydrolysis of 3′,5′-cyclic adenosine monophosphate (cAMP) and 3′,5′-cyclic guanosine monophosphate (cGMP)^7^. PDE4, PDE7, and PDE8 are specific for hydrolysis of cAMP. PDE5, PDE6, and PDE9 are specific for hydrolysis of cGMP. PDE1, PDE2, PDE3, PDE10, and PDE11 are non-specific and will hydrolyze both cAMP and cGMP^8^. Studies have shown that PDEs play an important role in the development of tumors by affecting the intracellular level of cAMP and/or cGMP and PDEs could become diagnostic markers or therapeutic targets^9^. PDE4 represents the greatest family, since it is constituted by 4 genes (PDE4A, PDE4B, PDE4C, PDE4D) differently distributed at tissue, cellular and subcellular levels, allowing different fine-tuned regulations^10^. Analysis of the mRNA expression of PDEs has revealed the expression of PDE4 in normal thyroid tissues^11^. Previous studies have also reported the increase of PDE4 activity in primary thyroid adenoma samples with mutant thyroid-stimulating hormone receptor (TSHR) or G alphas^12^. However, the potential role of PDE4 subtypes in THCA are still unclear and further research is needed.

In this study, we tried to elucidate the role of PDE4 subtypes by comprehensively analyzing the changes of PDE4 family in THCA. By using several bioinformatics databases, we first analyzed the expression levels of PDE4 subtypes in THCA patients, and evaluated the correlation between their expression levels and the prognosis of THCA. Next, the genetic alteration, methylation levels and the linkage between immune cell infiltration levels and PDE4 expression levels in THCA were explored. Finally, gene function enrichment analysis and protein–protein interaction (PPI) networks of PDE4 subtypes were investigated. Our research might help to clarify the regulatory role of PDE4 family in THCA, and provided a possibility to use PED4 family as potential biomarkers and new targets for THCA therapy.

## Materials and methods

### TCGA and GEO data acquisition

The clinical information and gene expression profiles of the TCGA-THCA cohort (59 normal and 505 tumor tissues) were obtained from the Cancer Genome Atlas (TCGA) database (https://portal.gdc.cancer.gov/). In the TCGA-THCA cohort, 505 tumor tissues contained 59 paired tumor tissues that matched normal tissues derived from the same THCA patients. The dataset (GSE153659) from the NCBI Gene Expression Omnibus (GEO) public database (https://www.ncbi.nlm.nih.gov/geo/), including 7 normal and 24 tumor samples, was used for the subsequent validation. R software and Bioconductor packages were used to compare the expression of PDE4 family between normal tissues and THCA tissues in the TCGA and GSE153659 datasets.

### GSCA analysis

Gene Set Cancer Analysis (GSCA, http://bioinfo.life.hust.edu.cn/GSCA/#/) is an integrated database for cancer gene set analysis at genomic, pharmacogenomic and immunogenomic levels^13,14^. The possible correlation between PDE4 family expression and progress free survival (PFS) in THCA were evaluated in the GSCA database.

### TNMplot analysis

TNMplot (http://www.tnmplot.com/) is a web application to compare gene expression changes between normal, primary tumor and metastatic tumor tissues^15^. Base on the RNA sequencing datasets of THCA patients from TCGA and the Genotype-Tissue Expression (GTEx) databases, the expression levels of PDE4 family were compared and visualized by boxplot, respectively.

### cBioPortal analysis

The cBioPortal (http://cbioportal.org) provides a web resource for exploring, visualizing and analyzing multidimensional cancer genomics data^16^. In this study, we conducted genetic alteration analysis of PDE4 family in THCA datasets by using the cBioPortal database.

### DiseaseMeth analysis

The Human Disease Methylation Database Version 3.0 (DiseaseMeth 3.0, http://diseasemeth.edbc.org/) is a web-based resource that focuses on the abnormal methylome of human diseases^17^. We used this database to explore the methylation levels of PDE4 family in THCA.

### TIMER analysis

Tumor Immune Estimation Resource (TIMER, https://cistrome.shinyapps.io/timer/) is a web comprehensive resource for the systematical analysis of tumor-infiltrating immune cells across different cancer types^18^. The TIMER database was used to analyze the effect of PDE4 family on the abundance of immune cells in THCA patients.

### Metascape analysis

Metascape (https://metascape.org/) is a web-based portal designed to provide a comprehensive gene list annotation and analysis resource^19^. It incorporates a core set of default ontologies for enrichment analysis, including Gene Ontology (GO) processes, Kyoto Encyclopedia of Genes and Genomes (KEGG) pathways, Reactome gene sets, canonical pathways, and the comprehensive resource of mammalian protein complexes (CORUM). The functional enrichment analysis of PDE4 family was performed using Metascape, with *P*-value less than 0.05 as the cutoff criterion.

### STRING analysis

STRING (https://cn.string-db.org/cgi/input.pl) is an online database for predicting PPI networks^20^. This database contains protein–protein interaction data, which can be either direct physical interaction or indirect functional correlation. The related signal proteins of PDE4 family were explored using STRING. Only protein–protein pairs with a high confidence score more than 0.7 were included for subsequent analysis.

## Results

### The expression of PDE4 family in THCA

**Figure 1:**
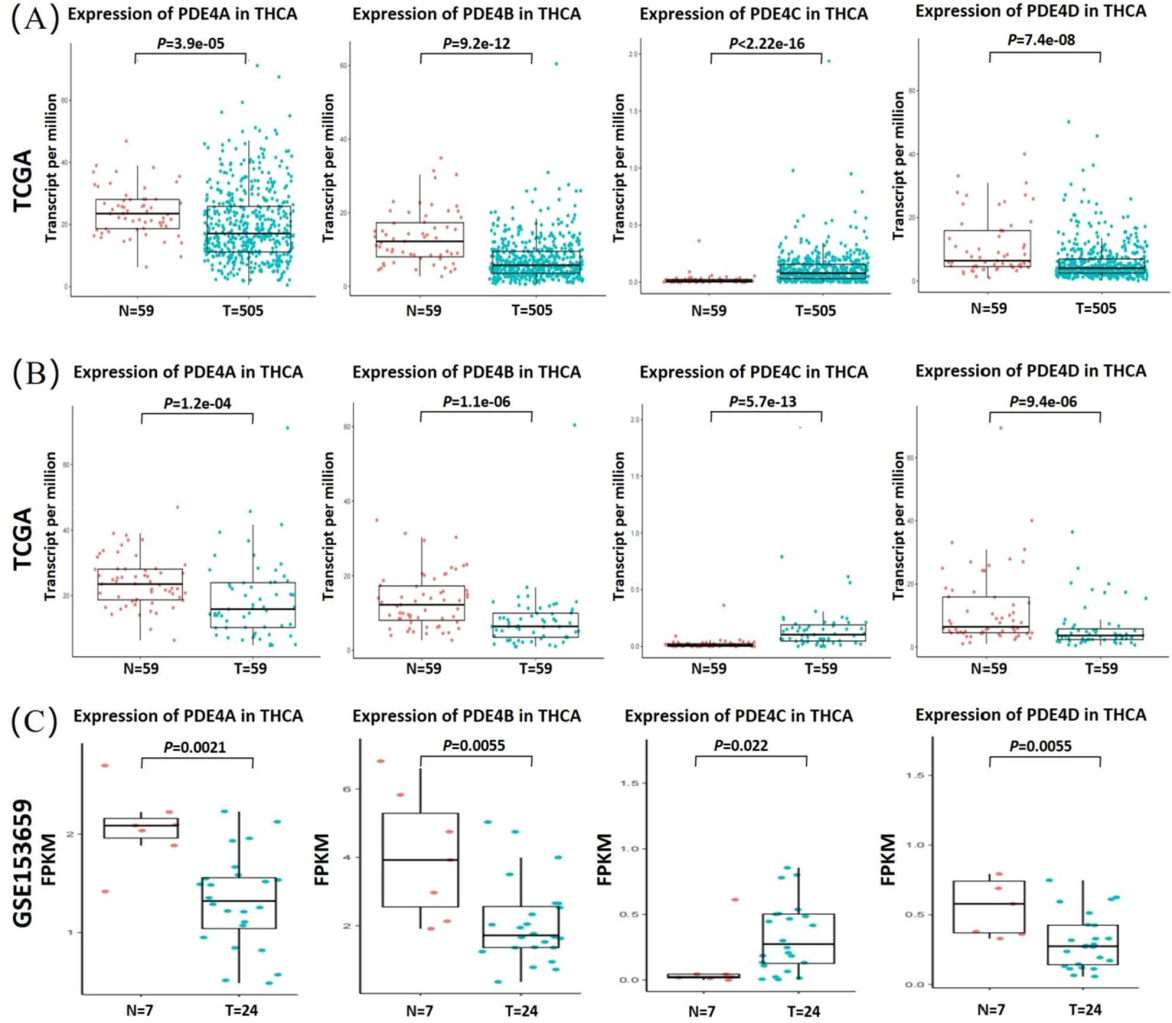
The expression of PDE4 family in thyroid carcinoma (THCA). (**A**) Base on the TCGA-THCA cohort, the mRNA expression profiles of four PDE4 subtypes were compared between 505 THCA tissues and 59 normal tissues; (**B**) The expression of four PDE4 subtypes were furtherly compared between 59 paired tumor tissues and 59 normal tissues in the TCGA-THCA cohort; (**C**) The expression of four PDE4 subtypes were verified in the GSE153659 dataset.

To better understand the functions of PDE4 family in THCA, TCGA datasets were used to evaluate the mRNA expression levels of PDE4A, PDE4B, PDE4C and PDE4D in THCA patients. The transcripts per million (TPM) expression values of PDE4 family were analyzed in 59 normal tissues and 505 primary THCA tissues (Fig. 1A). The expression differences of PDE4 family were further compared between 59 normal tissues and 59 paired tumor tissues in the TCGA-THCA cohort (Fig. 1B). These results showed that the expression levels of PDE4A, PDE4B and PDE4D were down-regulated in THCA tissues, while the expression level of PDE4C was significantly up-regulated. To further validate PDE4 family expression in THCA, we analyzed the cohort of 24 THCA tissues and 7 normal tissues from the GSE153659 dataset by using FPKM (fragments per kilobase of transcript per million) to quantify RNA-Seq data. Similarly, PDE4C was significantly up-regulated in THCA tissues, while other PDE4 subtypes were significantly down-regulated (Fig. 1C). These results suggested that PDE4C and other PDE4 subtypes might be have different expression regulation mechanisms in THCA tumorigenesis. All the clinical parameters of THCA patients involved were shown in Table 1. There were no differences in relevant parameters such as age, gender, and histology between the TGCA and GEO datasets.

**Table 1:** Baseline characteristics of THCA patients.

Patient characteristics	Database			*P Value*
	TCGA n = 505	TCGA (paired sample) n = 59	GEO (GSE153659) n = 24	
Age (years)				1.000
Median	47.27	45.86	42.79	
Range	15–89	15–79	28–65	
Sex, No				0.483
Male	136	14	4	
Female	369	45	20	
Histology, No				0.079
Follicular adenocarcinoma	1	1	0	
Follicular thyroid carcinoma	1	0	0	
Nonencapsulated sclerosing carcinoma	4	0	0	
Oxyphilic adenocarcinoma	1	0	0	
Papillary thyroid carcinoma (PTC)	356	48	24	
Follicular variant of papillary thyroid carcinoma (FVPTC)	104	10	0	
Columnar cell variant of papillary thyroid carcinoma (CCVPTC)	38	0	0	
Pathologic stage, No				*NA*
Stage I	284	36	0	
Stage II	52	9	0	
Stage III	112	12	0	
Stage IV	55	2	0	
Not reported	2	0	24	

NA: Not applicable.

The GSE153659 dataset lacked the information of pathologic stage.

### Relationship between PDE4 family expression and the prognosis of THCA

**Figure 2:**
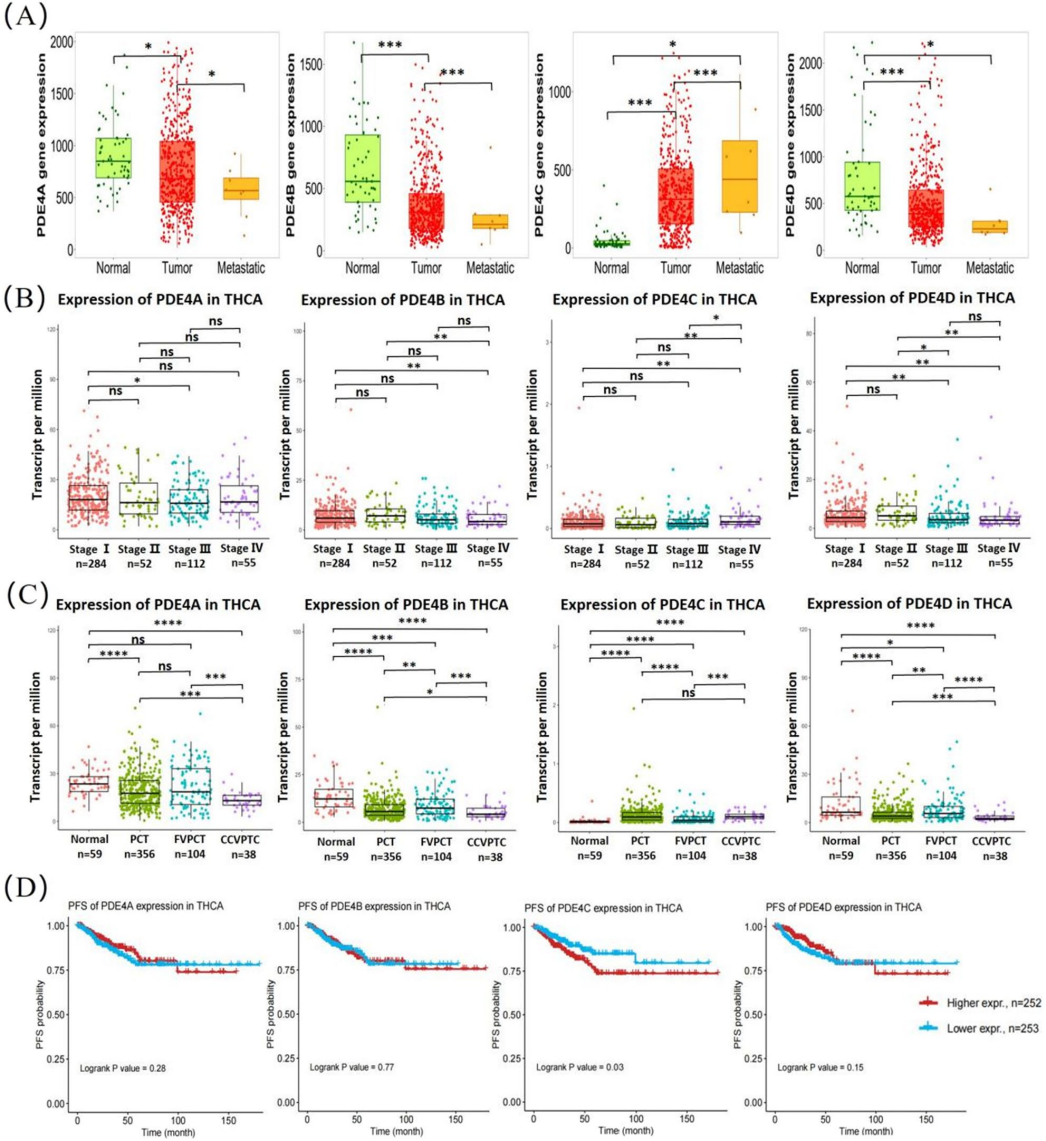
The prognostic value of PDE4 family in THCA patients. (**A**) The expression of four PDE4 subtypes were identified from TNMplot database on the cohort of 58 normal control, 502 primary and 8 metastatic THCA tissues; (**B**) Base on the TCGA-THCA cohort, the relationship between four PDE4 subtypes expression and THCA tissues with different pathological stages were analyzed; (**C**) The expression of four PDE4 subtypes in different subtypes of thyroid cancer were analyzed from the TCGA-THCA cohort of 59 normal tissues, 356 classical papillary thyroid carcinoma (PTC), 104 follicular variant of papillary thyroid carcinoma (FVPTC) and 38 columnar cell variant of papillary thyroid carcinoma (CCVPTC); (**D**) Though GSCA database, Kaplan–Meier survival curves were plotted to compare the survival difference between THCA patients with high PDE4 subtypes expression and THCA patients with low PDE4 subtypes expression. ns: not significant, *P ˂ 0.05, **P ˂ 0.01, ***P ˂ 0.001, ****P ˂ 0.0001.

To further elucidate the clinical significance of PDE4 family in THCA patients, we firstly evaluated the mRNA expression levels of PDE4 family in normal tissues (n = 58), primary THCA tissues (n = 502) and metastatic THCA tissues (n = 8) using the TNMplot database. Expression differences of PDE4 family were further validated between normal tissues and THCA tissues. We also found higher expression of PDE4C and lower expression of PDE4A, PDE4B and PDE4D in metastatic THCA tissues than those in primary THCA tissues (Fig. 2A). The results implied that the expression levels of PDE4 family were likely correlated with metastasis of THCA. In addition, based on the TCGA-THCA cohort, the mRNAs expression levels of PDE4 family were analyzed in different pathological stages of THCA. We found that the PDE4A expression level in cancer specimens obtained from 110 patients with stage III THCA was lower than that in 284 patients with stage I THCA. Similarly, patients with late-stage THCA tended to down-regulate the expression levels of PDE4B and PDE4D. However, up-regulated PDE4C was positively associated with THCA progression (Fig. 2B). To investigate the impact of the PDE4 family on different subtypes of THCA, we analyzed the mRNA expression levels of PDE4 family in normal tissues (n = 59), classical papillary thyroid carcinoma (PTC) (n = 356), follicular variant of papillary thyroid carcinoma (FVPTC) (n = 104) and columnar cell variant of papillary thyroid carcinoma (CCVPTC) (n = 38) using the TCGA-THCA cohort. We found that the patients with more aggressive forms of papillary thyroid carcinoma, such as CCVPTC, exhibited up-regulated PDE4C expression and down-regulated PDE4A/B/D expression compared with PTC and FVPTC (Fig. 2C). Finally, we assessed the prognostic value of PDE4 family expression in THCA patients from GSCA database. The results showed that THCA patients with higher PDE4C expression had shorter PFS than those with lower PDE4C expression (*P* = 0.03). However, the expression levels of other PDE4 subtypes had no effect on the survival of THCA patients (Fig. 2D). These data above indicated that PDE4C might be a diagnostic biomarker for the THCA prognosis.

### Genetic alteration and methylation of PDE4 family in THCA

**Figure 3:**
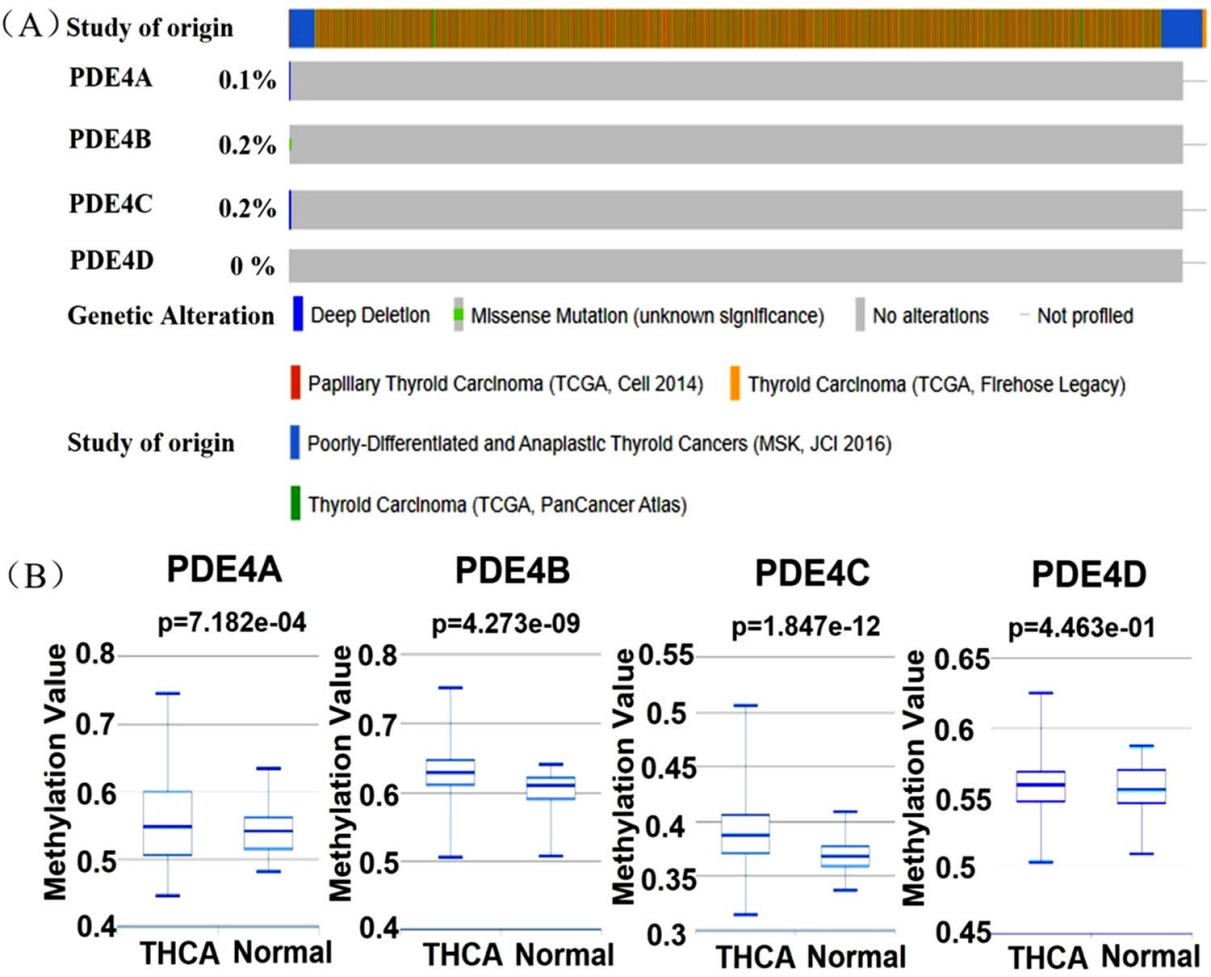
Genetic alteration and methylation of PDE4 family in THCA patients. (**A**) Genetic alteration of four PDE4 subtypes in THCA patients from cBioPortal database; (**B**) The methylation levels of four PDE4 subtypes in THCA patients from DiseaseMeth database.

Genetic alteration is a pivotal factor resulting in tumor progression^21^. We used the cBioPortal database to analyze genetic alterations of PDE4 family in THCA patients (1620 patients from four datasets). The genetic alteration frequency of PDE4A, PDE4B, PDE4C and PDE4D was 0.1%, 0.2%, 0.2% and 0%, respectively (Fig. 3A). The type of genetic alteration contained deep deletion and missense mutation. Gene methylation is recognized as another important factor in tumor progression^22^. Methylation analysis of PDE4 family was performed in THCA tissues and normal tissues from DiseaseMeth website. And the results showed that the methylation levels of PDE4A (*P* = 7.182e − 04), PDE4B (*P* = 4.273e − 09) and PDE4C (*P* = 1.847e − 12) were higher in THCA tissues than that in normal tissues. But the methylation level of PDE4D had no significant change between THCA tissues and normal tissues (Fig. 3B). These results hinted that genetic alteration of PDE4 family might not be the important factor in THCA tumorigenesis.

### The correlation between immune cell infiltration and PDE4 family in THCA

**Figure 4:**
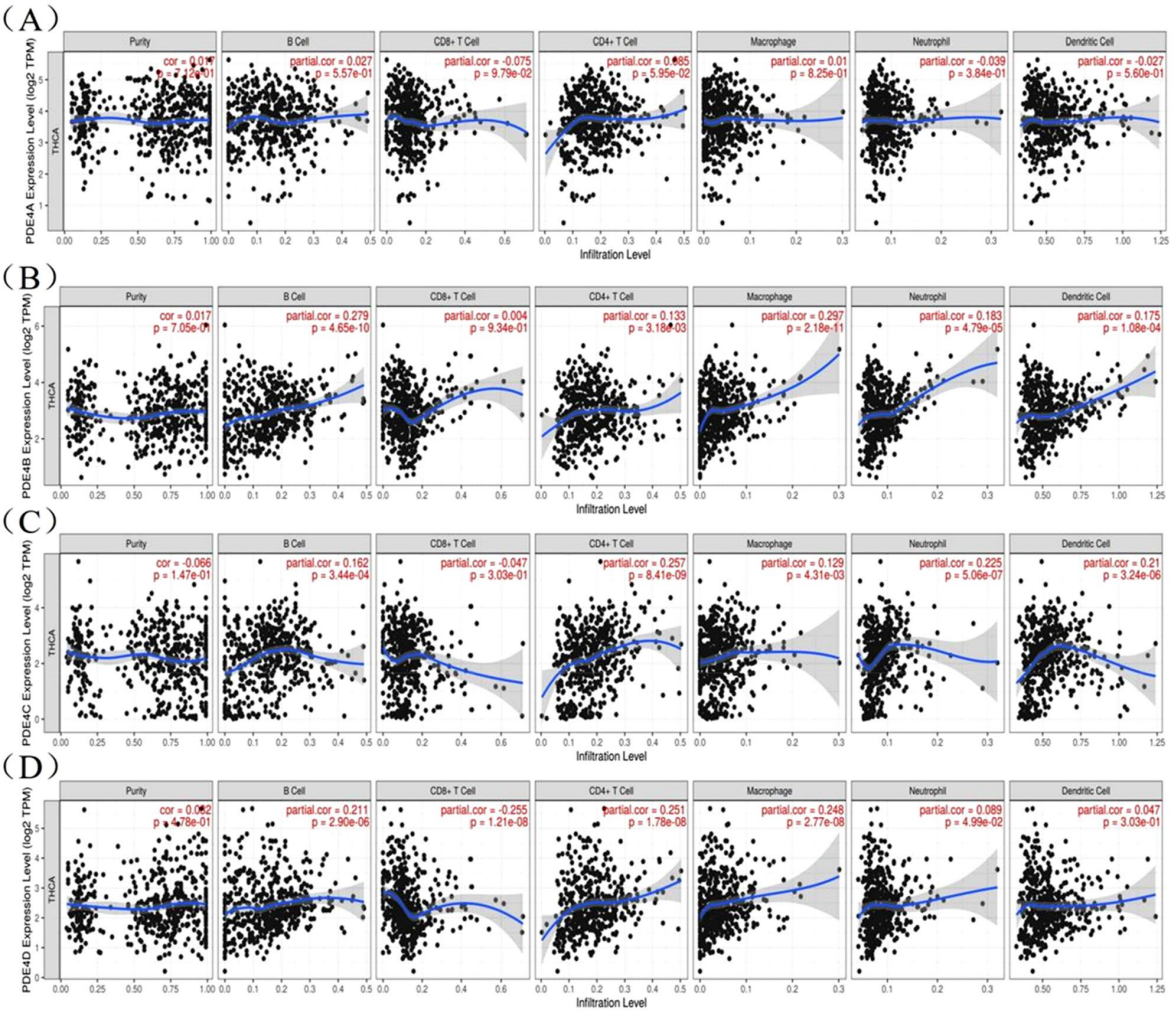
The correlation between immune cell infiltration levels and PDE4 family expression in THCA from TIMER database. (**A**) PDE4A; (**B**) PDE4B; (**C**) PDE4C; (**D**) PDE4D.

Tumor-infiltrating lymphocytes (TILs) are an integral component of the tumor microenvironment and have been found to correlate with prognosis of cancer^23^. Therefore, we assessed the relationship between PDE4 family expression and six distinct tumor infiltrating immune subsets from TIMER database. The results showed that PDE4A expression had no significant effect on the infiltration levels of six immune cells in THCA (Fig. 4A). However, PDE4B expression was positively linked to the infiltrated B cell (Cor = 0.279, *P* = 4.65e − 10), CD4^+^ T cell (Cor = 0.133, *P* = 3.18e − 03), macrophage (Cor = 0.279, *P* = 2.18e − 11), neutrophil (Cor = 0.183, *P* = 4.79e − 05) and dendritic cell (Cor = 0.175, *P* = 1.08e − 04) in THCA (Fig. 4B). PDE4C was positively linked to the infiltrated B cell (Cor = 0.162, *P* = 3.44e − 04), CD4^+^ T cell (Cor = 0.257, *P* = 8.41e − 09), macrophage (Cor = 0.129, *P* = 4.13e − 03), neutrophil (Cor = 0.225, *P* = 5.06e − 07), dendritic cell (Cor = 0.21, *P* = 3.24e − 06) in THCA (Fig. 4C). PDE4D was positively linked to the infiltrated B cell (Cor = 0.211, *P* = 2.90e − 06), CD4^+^ T cell (Cor = 0.251, *P* = 1.78e − 08), macrophage (Cor = 0.248, *P* = 2.77e − 08), neutrophil (Cor = 0.089, *P* = 4.99e − 02), but negatively linked to the CD8^+^ T cell (Cor = − 0.255, *P* = 1.21e − 08) in THCA (Fig. 4D). These results implied that, except for PDE4A, PDE4B, PDE4C and PDE4D could affect immune cells infiltration in the progression of THCA.

### Functional enrichment analysis and PPI networks of PDE4 family

**Figure 5:**
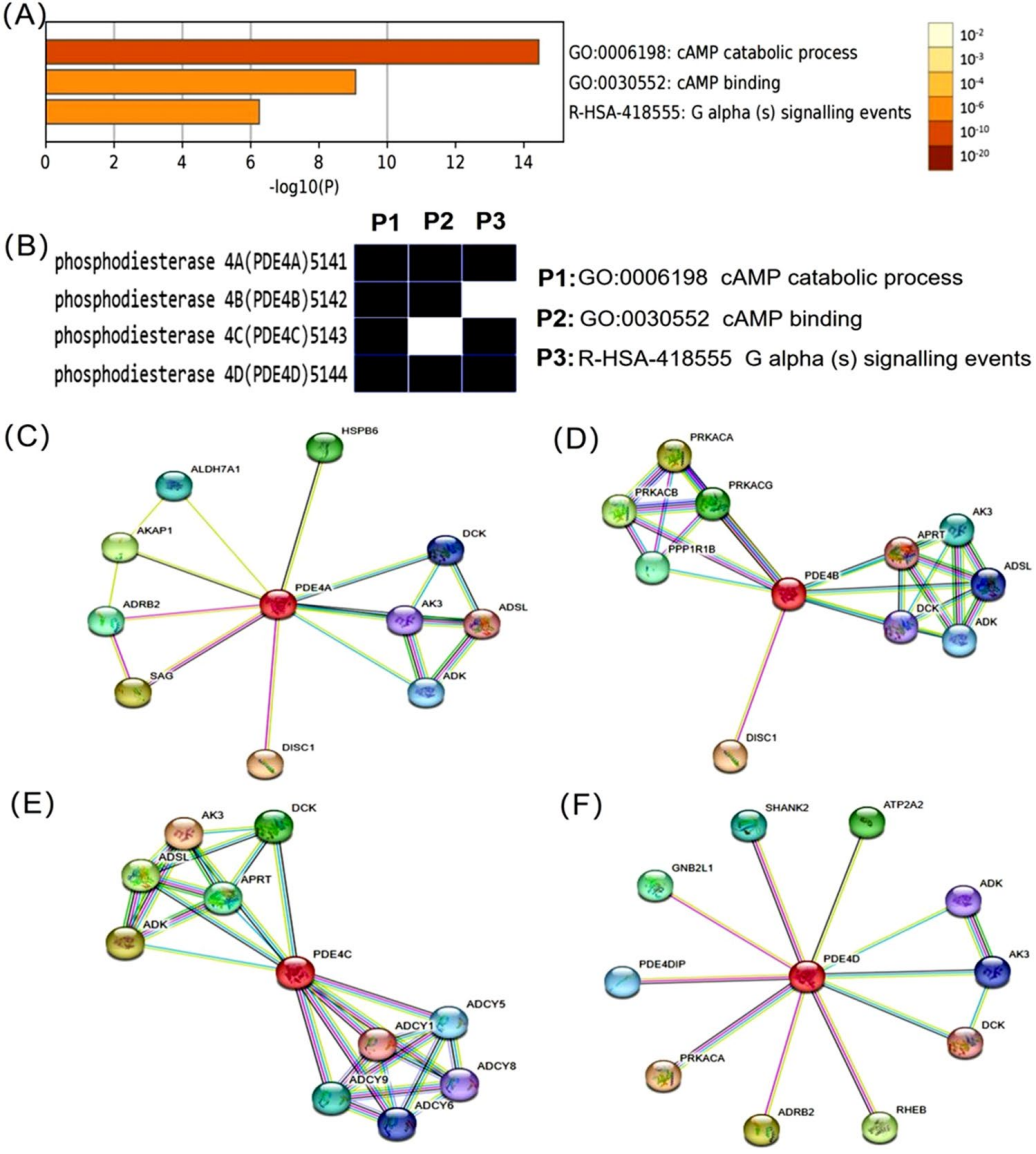
Functional enrichment analysis and PPI networks of PDE4 family. (**A**) The bar graph showed the enrichment analysis results of PDE4 family related signaling pathways from Metascape databases. One row per cluster, using a discrete color scale to represent statistical significance. (**B**) Functional enrichment heatmap of four PDE4 subtypes were collected from Metascape databases. (**C**–**F**) The PPI networks of four PDE4 subtypes were generated using STRING. Nodes represent proteins and edges represent protein–protein interactions. (**C**) PDE4A; (**D**) PDE4B; (**E**) PDE4C; (**F**) PDE4D.

To clarify the functions and signaling pathways of PDE4 family, we performed functional enrichment analysis of PDE4 family from Metascape database. Parameter setting was *P* < 0.05, minimum count of 3, and enrichment factor > 1.5. The enrichment results showed three potential PDE4 family related signaling pathways, including cAMP catabolic process (GO:0006198), cAMP binding (GO:0030552) and G alphas signaling events (R-HSA-418555) (Fig. 5A). We found that four PDE4 subtypes were all enriched in cAMP catabolic process. Nevertheless, PDE4C was not enriched in the cAMP binding signal pathway, and PDE4B was not enriched in the G alphas signaling events (Fig. 5B). In addition, we used STRING database to explore the PPI networks of PDE4 family. We found that PDE4A (Fig. 5C, Supplementary Table 1), PDE4B (Fig. 5D, Supplementary Table 2), PDE4C (Fig. 5E, Supplementary Table 3) and PDE4D (Fig. 5F, Supplementary Table 4) were all correlated with deoxycytidine kinase (DCK), adenosine kinase (ADK) and adenylate kinase 3(AK3). Besides, PDE4A was related to heat shock protein B6 (HSPB6), aldehyde dehydrogenase 7A1 (ALDH7A1), A-kinase anchoring protein 1 (AKAP1), adrenoceptor beta 2 (ADRB2), SAG (an arrestin family member which desensitizes GPCR) and DISC1 (disrupted in Schizophrenia 1). PDE4B was correlated with DISC1, adenylosuccinate lyase (ADSL), adenine phosphoribosyltransferase (APRT), protein phosphatase 1 regulatory inhibitor subunit 1B (PPP1R1B) and three catalytic subunit of protein kinase A (PPKACA, PPKACB and PPKACG). PDE4C was related to ADSL, APRT and five adenylate cyclases (ADCY1, ADCY5, ADCY6, ADCY8 and ADCY9). And PDE4D was related to ATPase sarcoplasmic/endoplasmic reticulum Ca^2+^ transporting 2 (ATP2A2), SH3 and multiple ankyrin repeat domains 2 (SHANK2), guanine nucleotide-binding protein, beta polypeptide 2-like 1 (GNB2L1), PDE4D interacting protein (PDE4DIP), PPKACA, ADRB2 and Ras homolog protein enriched in brain (Rheb). These results indicated that, in addition to the role of catabolizing cAMP, four PDE4 subtypes have highly specific and non-redundant functions.

## Discussion

Our study found that PDE4A, PDE4B and PDE4D were down-regulated in THCA tissues compared with normal tissues, and their low expression levels were related to the metastasis of THCA (Figs. 1, 2A). Similarly, PDE4A expression decreased in CD34^+^ granulocytes taken from patients with essential thrombocytopenia, polycythemia vera, and primary myelofibrosis^24^. PDE4B expression was also decreased in castration-resistant prostate cancer and advanced prostate cancer^25^. The down-regulated PDE4D was reported recently to promote the progression of prostate cancer and bladder cancer^26,27^. However, some researchers also provided different results, such as over-expression of PDE4A, PDE4B and PDE4D in cancers of the lung and breast^28^. It was reported that the expression of PDE4 family was regulated by mitogen-activated protein kinase signaling pathways, such as extracellular signal-regulated kinase (ERK) and MAPK-activated protein kinase 2 (MK2)^29–31^. As we know, up to 70% of THCA were caused by mutations that activate the ERK signaling pathway^32^, and MK2 was strongly expressed in papillary thyroid carcinomas and follicular thyroid carcinomas cells^33^. Therefore, we speculated that down-regulated expression of PDE4A, PDE4B and PDE4D in THCA might be related to enhanced ERK or MK2 signaling pathway. To some extent, the hypothesis could be supported by the enhancement of ERK or MK2 signaling pathways in cancers, such as prostate cancer and bladder cancer, which presented down-regulation of PDE4 expression^34,35^.

By analyzing TCGA and GEO (GSE153659) datasets, we found that PDE4C was significantly up-regulated in THCA tissues (Fig. 1). Moreover, THCA patients with higher PDE4C expression had shorter PFS compared with those with lower PDE4C expression (Fig. 2). These results implied that up-regulated PDE4C might be a potential diagnostic marker of THCA and a target for inhibiting THCA progression. Due to the trace expression of PDE4C in most tissues and organs, there is paucity of data to elucidate PDE4C-mediated oncogenesis and related mechanism^36,37^. In our results, PDE4C expression exhibited an opposite trend compared to other PDE4 subtypes. Up-regulation of PDE4C expression was accompanied by down-regulation of PDE4A, PDE4B and PDE4D. It seems that PDE4C and other PDE subtypes play different roles in the THCA process, and up-regulation of PDE4C can accelerate THCA progression. This discovery focused our attention on PDE4 inhibitors. Indeed, the research field of PDE4 inhibitors was very active, including inflammation-based diseases, autoimmune disease and cancers^38,39^. But the research of PDE4 inhibitors in cancer mainly surround PDE4A, PDE4B, and PDE4D^28^. Our study here supported PDE4C inhibitor as a potential drug of THCA, and it might open up the possibility of PDE4C related research across malignancies.

**Figure 6:**
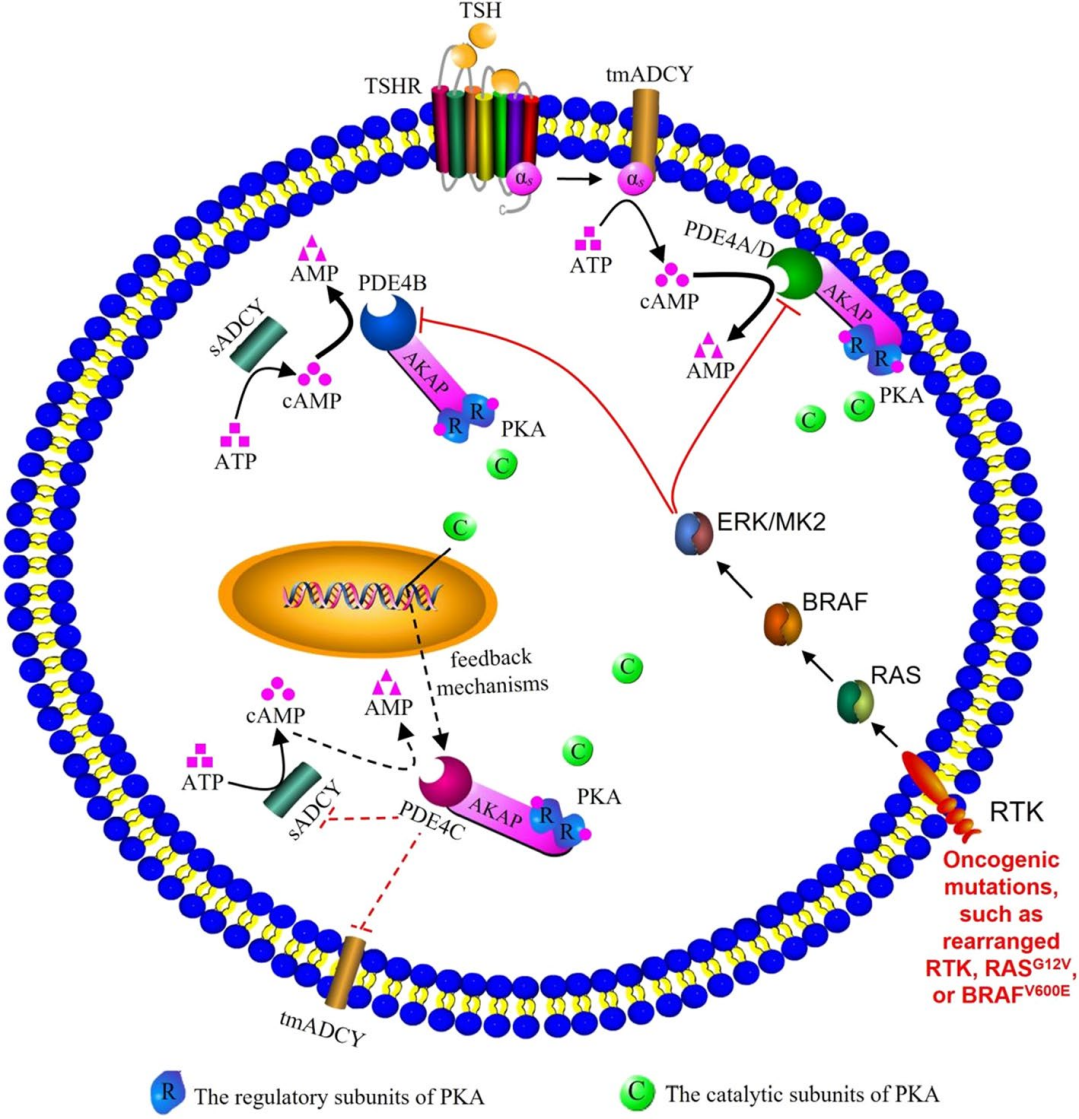
The schematic diagram of PDE4 family in THCA cells. TSH, Thyroid-stimulating hormone; TSHR, TSH receptor; tmADCY, Transmembrane adenylate cyclase; sADCY, Soluble adenylate cyclase; ATP, Adenosine triphosphate; cAMP, Cyclic adenosine monophosphate; AMP, Adenosine monophosphate; RTK, Receptor tyrosine kinases; PKA, Protein kinase A; AKAP, A-kinase anchoring protein; ERK, Extracellular signal- regulated kinases; MK2, MAPK-activated protein kinase 2; R, The regulatory subunits of PKA; C, The catalytic subunits of PKA. The black lines mean stimulation, and red lines mean inhibitory effect. The solid arrows mean that the relevant effects have been supported by the literature, and dotted arrows mean that the relevant effects are the conjectures of this study.

The cAMP pathway plays a important role in growth regulation of thyroid cells and thyroid tumorigenesis^40^. Based on literatures and results of this study, we attempted to draw a schematic diagram to display the possible mechanisms by which the PDE4 family regulates cAMP related signaling pathways in THCA cell (Fig. 6). Notably elevated thyroid-stimulating hormone (TSH) binds to the TSHR and couples preferentially to the G alphas (α_s_) protein, resulting in ADCY activation and an increase of cAMP level^41^. Increased cAMP leads to activation of A-kinase anchoring protein (AKAP) bound protein kinase A (PKA), which in turn phosphorylates PDE4, thereby terminating cAMP-PKA signaling^42,43^. PDE4A and PDE4D participate in G alpha signaling events (Fig. 5B), and may be responsible for regulating local cAMP level near plasma membrane and limiting the activation of transmembrane ADCY (tmADCY) -associated cAMP-PKA signaling (Fig. 5, Supplementary Table 1, 4)^29,42,44,45^. Oppositely, PDE4B does not participate in G alphas signaling events (Fig. 5B), therefore, may inhibit the activation of soluble ADCY (sADCY) -associated cAMP-PKA signaling in the cytoplasm (Fig. 5D, Supplementary Table 2)^46,47^. In THCA, ERK or MK2 signaling is usually activated by oncogenic mutations such as rearranged RTK, RAS^G12V^, or BRAF^V600E^^32,33^, and subsequently mediates inhibition of PDE4A, PDE4B and PDE4D^29–31^. The limitation of the three PDE4 subtypes lead to an increase in cAMP activated PKA, which may gradually up-regulate the expression of PDE4C through some unknown feedback mechanisms (Figs. 1, 2A,B). However, PDE4C does not involve in the cAMP binding process (Fig. 5B), so we speculate that the cAMP binding affinity of PDE4C was lower than that of other PDE4 subtypes, which weakens its ability to hydrolyze cAMP^48^. Up-regulated PDE4C may reduce cAMP signaling by inhibiting ADCYs that catalyze the generation of cAMP, rather than directly hydrolyzing cAMP, ultimately leading to the progression of THCA (Fig. 5B,E)^48–52^.

In conclusion, we systematically analyzed the PDE4 family in THCA patients, involving the expression, prognosis and gene regulation network. Although the results observed from several bioinformatics databases to some extent alleviate the strength of the relevant conclusions. The consistent trend of PDE4 family changes in different bioinformatics databases provides a theoretical basis for studying the regulatory mechanism of cAMP signaling pathways in THCA. In addition, our research suggests that PDE4C may become a potential prognostic candidate and supports specific PDE4C inhibitors as therapeutic drugs for THCA.

### Supplementary Information

Supplementary Tables.

## Data Availability

The original contributions presented in the study are included in the article/supplementary material, further inquiries can be directed to the corresponding authors.
