# Supplementary material for: Elevated PDE4C level serves as a candidate diagnostic biomarker and correlates with poor survival in thyroid carcinoma
Source: Sci Rep. 2024 Mar 21;14:6813. doi: 10.1038/s41598-024-57533-w (PMC10957934; doi:10.1038/s41598-024-57533-w)
Supplement: Supplementary file 1 — Supplementary Tables. [file 41598_2024_57533_MOESM1_ESM.pdf]

Supplementary Table 1. The enrichment process of PDE4A.

#term ID	term description	observed gene count	background gene count	strength	false discovery rate	matching proteins in your network (IDs)	matching proteins in your network (labels)
GO:0009126	Purine nucleoside monophosphate metabolic process	4	39	2.26	8.46E-05	9606.ENSP00000286621,9606.ENSP00000286648,9606.ENSP00000371230,9606.ENSP00000485525	ADK,DCK,AK3,ADSL
GO:0046033	AMP metabolic process	3	14	2.58	0.00044	9606.ENSP00000286621,9606.ENSP00000371230,9606.ENSP00000485525	ADK,AK3,ADSL
GO:0009127	Purine nucleoside monophosphate biosynthetic process	3	21	2.41	0.00085	9606.ENSP00000286621,9606.ENSP00000286648,9606.ENSP00000485525	ADK,DCK,ADSL
GO:0006163	Purine nucleotide metabolic process	5	329	1.43	0.0014	9606.ENSP00000286621,9606.ENSP00000286648,9606.ENSP00000370078,9606.ENSP00000371230,9606.ENSP00000485525	ADK,DCK,PDE4A,AK3,ADSL
GO:0010738	Regulation of protein kinase a signaling	3	27	2.3	0.0014	9606.ENSP00000305372,9606.ENSP00000370078,9606.ENSP00000478212	ADRB2,PDE4A,AKAP1
GO:0006164	Purine nucleotide biosynthetic process	4	158	1.65	0.002	9606.ENSP00000286621,9606.ENSP00000286648,9606.ENSP00000371230,9606.ENSP00000485525	ADK,DCK,AK3,ADSL
GO:0006167	AMP biosynthetic process	2	5	2.85	0.0048	9606.ENSP00000286621,9606.ENSP00000485525	ADK,ADSL
GO:0009153	Purine deoxyribonucleotide biosynthetic process	2	5	2.85	0.0048	9606.ENSP00000286621,9606.ENSP00000286648	ADK,DCK
GO:0009150	Purine ribonucleotide metabolic process	4	303	1.37	0.0115	9606.ENSP00000286621,9606.ENSP00000370078,9606.ENSP00000371230,9606.ENSP00000485525	ADK,PDE4A,AK3,ADSL
GO:0009116	Nucleoside metabolic process	3	104	1.71	0.0144	9606.ENSP00000286621,9606.ENSP00000286648,9606.ENSP00000371230	ADK,DCK,AK3
GO:0034404	Nucleobase-containing small molecule biosynthetic process	3	105	1.71	0.0144	9606.ENSP00000286621,9606.ENSP00000286648,9606.ENSP00000370078	ADK,DCK,PDE4A
GO:0043101	Purine-containing compound salvage	2	15	2.38	0.0178	9606.ENSP00000286621,9606.ENSP00000286648	ADK,DCK
GO:0043174	Nucleoside salvage	2	16	2.35	0.0194	9606.ENSP00000286621,9606.ENSP00000286648	ADK,DCK
GO:0002029	Desensitization of g protein-coupled receptor signaling pathway	2	17	2.32	0.0197	9606.ENSP00000305372,9606.ENSP00000386444	ADRB2,SAG
GO:0009152	Purine ribonucleotide biosynthetic process	3	147	1.56	0.0242	9606.ENSP00000286621,9606.ENSP00000371230,9606.ENSP00000485525	ADK,AK3,ADSL
GO:0044271	Cellular nitrogen compound biosynthetic process	6	1522	0.85	0.0248	9606.ENSP00000286621,9606.ENSP00000286648,9606.ENSP00000370078,9606.ENSP00000371230,9606.ENSP00000387123,9606.ENSP00000485525	ADK,DCK,PDE4A,AK3,ALDH7A1,ADSL
GO:1901135	Carbohydrate derivative metabolic process	5	987	0.95	0.0371	9606.ENSP00000286621,9606.ENSP00000286648,9606.ENSP00000370078,9606.ENSP00000371230,9606.ENSP00000485525	ADK,DCK,PDE4A,AK3,ADSL
GO:0034654	Nucleobase-containing compound biosynthetic process	5	995	0.95	0.0376	9606.ENSP00000286621,9606.ENSP00000286648,9606.ENSP00000370078,9606.ENSP00000371230,9606.ENSP00000485525	ADK,DCK,PDE4A,AK3,ADSL
GO:0044281	Small molecule metabolic process	6	1684	0.8	0.0387	9606.ENSP00000286621,9606.ENSP00000286648,9606.ENSP00000370078,9606.ENSP00000371230,9606.ENSP00000387123,9606.ENSP00000485525	ADK,DCK,PDE4A,AK3,ALDH7A1,ADSL

Supplementary Table 2. The enrichment process of PDE4B.

#term ID	term description	observed gene count	background gene count	strength	false discovery rate	matching proteins in your network (IDs)	matching proteins in your network (labels)
GO:0009126	Purine nucleoside monophosphate metabolic process	5	39	2.36	2.66E-07	9606.ENSP00000286621,9606.ENSP00000286648, 9606.ENSP00000367615,9606.ENSP00000371230, 9606.ENSP00000485525	ADK,DCK,APRT,AK3,ADSL
GO:0046033	AMP metabolic process	4	14	2.71	1.06E-06	9606.ENSP00000286621,9606.ENSP00000367615, 9606.ENSP00000371230,9606.ENSP00000485525	ADK,APRT,AK3,ADSL
GO:0009127	Purine nucleoside monophosphate biosynthetic process	4	21	2.53	2.18E-06	9606.ENSP00000286621,9606.ENSP00000286648, 9606.ENSP00000367615,9606.ENSP00000485525	ADK,DCK,APRT,ADSL
GO:0006167	AMP biosynthetic process	3	5	3.03	1.58E-05	9606.ENSP00000286621,9606.ENSP00000367615, 9606.ENSP00000485525	ADK,APRT,ADSL
GO:0006163	Purine nucleotide metabolic process	6	329	1.51	1.74E-05	9606.ENSP00000286621,9606.ENSP00000286648, 9606.ENSP00000332116,9606.ENSP00000367615, 9606.ENSP00000371230,9606.ENSP00000485525	ADK,DCK,PDE4B,APRT,AK3,ADSL
GO:0006164	Purine nucleotide biosynthetic process	5	158	1.75	2.38E-05	9606.ENSP00000286621,9606.ENSP00000286648, 9606.ENSP00000367615,9606.ENSP00000371230, 9606.ENSP00000485525	ADK,DCK,APRT,AK3,ADSL
GO:0034380	High-density lipoprotein particle assembly	3	13	2.61	6.31E-05	9606.ENSP00000309591,9606.ENSP00000359719, 9606.ENSP00000366488	PRKACA,PRKACB,PRKACG
GO:0006796	Phosphate-containing compound metabolic process	9	2107	0.88	7.14E-05	9606.ENSP00000286621,9606.ENSP00000286648, 9606.ENSP00000309591,9606.ENSP00000332116, 9606.ENSP00000359719,9606.ENSP00000366488, 9606.ENSP00000367615,9606.ENSP00000371230, 9606.ENSP00000485525	ADK,DCK,PRKACA,PDE4B,PRKACB,PRKACG,APRT,AK3,ADSL
GO:0010737	Protein kinase a signaling	3	15	2.55	7.65E-05	9606.ENSP00000309591,9606.ENSP00000359719, 9606.ENSP00000366488	PRKACA,PRKACB,PRKACG
GO:0043101	Purine-containing compound salvage	3	15	2.55	7.65E-05	9606.ENSP00000286621,9606.ENSP00000286648, 9606.ENSP00000367615	ADK,DCK,APRT
GO:0043174	Nucleoside salvage	3	16	2.52	7.65E-05	9606.ENSP00000286621,9606.ENSP00000286648, 9606.ENSP00000367615	ADK,DCK,APRT
GO:0034199	Activation of protein kinase a activity	3	19	2.45	0.00011	9606.ENSP00000309591,9606.ENSP00000359719, 9606.ENSP00000366488	PRKACA,PRKACB,PRKACG
GO:0034654	Nucleobase-containing compound biosynthetic process	7	995	1.1	0.00012	9606.ENSP00000254079,9606.ENSP00000286621, 9606.ENSP00000286648,9606.ENSP00000332116, 9606.ENSP00000367615,9606.ENSP00000371230, 9606.ENSP00000485525	PPP1R1B,ADK,DCK,PDE4B,APRT,AK3,ADSL
GO:0009116	Nucleoside metabolic process	4	104	1.84	0.00013	9606.ENSP00000286621,9606.ENSP00000286648, 9606.ENSP00000367615,9606.ENSP00000371230	ADK,DCK,APRT,AK3
GO:0034404	Nucleobase-containing small molecule biosynthetic process	4	105	1.83	0.00013	9606.ENSP00000286621,9606.ENSP00000286648, 9606.ENSP00000332116,9606.ENSP00000367615	ADK,DCK,PDE4B,APRT
GO:0009150	Purine ribonucleotide metabolic process	5	303	1.47	0.00017	9606.ENSP00000286621,9606.ENSP00000332116, 9606.ENSP00000367615,9606.ENSP00000371230, 9606.ENSP00000485525	ADK,PDE4B,APRT,AK3,ADSL
GO:0071377	Cellular response to glucagon stimulus	3	27	2.3	0.00021	9606.ENSP00000309591,9606.ENSP00000359719, 9606.ENSP00000366488	PRKACA,PRKACB,PRKACG
GO:0009152	Purine ribonucleotide biosynthetic process	4	147	1.68	0.00034	9606.ENSP00000286621,9606.ENSP00000367615, 9606.ENSP00000371230,9606.ENSP00000485525	ADK,APRT,AK3,ADSL
GO:0003091	Renal water homeostasis	3	37	2.16	0.00039	9606.ENSP00000309591,9606.ENSP00000359719, 9606.ENSP00000366488	PRKACA,PRKACB,PRKACG
GO:0044209	AMP salvage	2	2	3.25	0.00048	9606.ENSP00000286621,9606.ENSP00000367615	ADK,APRT
GO:0046128	Purine ribonucleoside metabolic process	3	58	1.96	0.0011	9606.ENSP00000286621,9606.ENSP00000367615, 9606.ENSP00000371230	ADK,APRT,AK3
GO:0050878	Regulation of body fluid levels	5	509	1.24	0.0012	9606.ENSP00000309591,9606.ENSP00000359719, 9606.ENSP00000366488,9606.ENSP00000367615, 9606.ENSP00000371230	PRKACA,PRKACB,PRKACG,APRT,AK3
GO:0006166	Purine ribonucleoside salvage	2	5	2.85	0.0014	9606.ENSP00000286621,9606.ENSP00000367615	ADK,APRT
GO:0009153	Purine deoxyribonucleotide biosynthetic process	2	5	2.85	0.0014	9606.ENSP00000286621,9606.ENSP00000286648	ADK,DCK
GO:0010243	Response to organonitrogen compound	6	987	1.03	0.0014	9606.ENSP00000254079,9606.ENSP00000309591, 9606.ENSP00000332116,9606.ENSP00000359719, 9606.ENSP00000366488,9606.ENSP00000367615	PPP1R1B,PRKACA,PDE4B,PRKACB,PRKACG,APRT
GO:1901135	Carbohydrate derivative metabolic process	6	987	1.03	0.0014	9606.ENSP00000286621,9606.ENSP00000286648, 9606.ENSP00000332116,9606.ENSP00000367615, 9606.ENSP00000371230,9606.ENSP00000485525	ADK,DCK,PDE4B,APRT,AK3,ADSL
GO:1901621	Negative regulation of smoothened signaling pathway involved in dorsal/ventral neural tube patterning	2	6	2.77	0.0017	9606.ENSP00000309591,9606.ENSP00000359719	PRKACA,PRKACB
GO:0071417	Cellular response to organonitrogen compound	5	590	1.18	0.002	9606.ENSP00000309591,9606.ENSP00000332116, 9606.ENSP00000359719,9606.ENSP00000366488, 9606.ENSP00000367615	PRKACA,PDE4B,PRKACB,PRKACG,APRT
GO:0071375	Cellular response to peptide hormone stimulus	4	269	1.42	0.0021	9606.ENSP00000309591,9606.ENSP00000359719, 9606.ENSP00000366488,9606.ENSP00000367615	PRKACA,PRKACB,PRKACG,APRT
GO:1901137	Carbohydrate derivative biosynthetic process	5	602	1.17	0.0021	9606.ENSP00000286621,9606.ENSP00000286648, 9606.ENSP00000367615,9606.ENSP00000371230, 9606.ENSP00000485525	ADK,DCK,APRT,AK3,ADSL
GO:0006139	Nucleobase-containing compound metabolic process	8	2659	0.73	0.0024	9606.ENSP00000254079,9606.ENSP00000286621, 9606.ENSP00000286648,9606.ENSP00000309591, 9606.ENSP00000332116,9606.ENSP00000367615, 9606.ENSP00000371230,9606.ENSP00000485525	PPP1R1B,ADK,DCK,PRKACA,PDE4B,APRT,AK3,ADSL
GO:0007596	Blood coagulation	4	303	1.37	0.0031	9606.ENSP00000309591,9606.ENSP00000359719, 9606.ENSP00000366488,9606.ENSP00000371230	PRKACA,PRKACB,PRKACG,AK3
GO:0002429	Immune response-activating cell surface receptor signaling pathway	4	311	1.36	0.0033	9606.ENSP00000309591,9606.ENSP00000332116, 9606.ENSP00000359719,9606.ENSP00000366488	PRKACA,PDE4B,PRKACB,PRKACG
GO:0002223	Stimulatory c-type lectin receptor signaling pathway	3	111	1.68	0.0046	9606.ENSP00000309591,9606.ENSP00000359719, 9606.ENSP00000366488	PRKACA,PRKACB,PRKACG
GO:0071872	Cellular response to epinephrine stimulus	2	13	2.44	0.0046	9606.ENSP00000309591,9606.ENSP00000332116	PRKACA,PDE4B
GO:1901700	Response to oxygen-containing compound	6	1567	0.83	0.0108	9606.ENSP00000254079,9606.ENSP00000309591, 9606.ENSP00000332116,9606.ENSP00000359719, 9606.ENSP00000366488,9606.ENSP00000367615	PPP1R1B,PRKACA,PDE4B,PRKACB,PRKACG,APRT
GO:1901701	Cellular response to oxygen-containing compound	5	1055	0.93	0.0186	9606.ENSP00000309591,9606.ENSP00000332116, 9606.ENSP00000359719,9606.ENSP00000366488, 9606.ENSP00000367615	PRKACA,PDE4B,PRKACB,PRKACG,APRT
GO:0006807	Nitrogen compound metabolic process	10	6852	0.41	0.0224	9606.ENSP00000254079,9606.ENSP00000286621, 9606.ENSP00000286648,9606.ENSP00000309591, 9606.ENSP00000332116,9606.ENSP00000359719, 9606.ENSP00000366488,9606.ENSP00000367615, 9606.ENSP00000371230,9606.ENSP00000485525	PPP1R1B,ADK,DCK,PRKACA,PDE4B,PRKACB,PRKACG,APRT,AK3,ADSL
GO:1901564	Organonitrogen compound metabolic process	9	5244	0.48	0.0242	9606.ENSP00000286621,9606.ENSP00000286648, 9606.ENSP00000309591,9606.ENSP00000332116, 9606.ENSP00000359719,9606.ENSP00000366488, 9606.ENSP00000367615,9606.ENSP00000371230, 9606.ENSP00000485525	ADK,DCK,PRKACA,PDE4B,PRKACB,PRKACG,APRT,AK3,ADSL
GO:0065008	Regulation of biological quality	8	4042	0.55	0.0309	9606.ENSP00000254079,9606.ENSP00000309591, 9606.ENSP00000332116,9606.ENSP00000355593, 9606.ENSP00000359719,9606.ENSP00000366488, 9606.ENSP00000367615,9606.ENSP00000371230	PPP1R1B,PRKACA,PDE4B,DISC1,PRKACB,PRKACG,APRT,AK3
GO:0006220	Pyrimidine nucleotide metabolic process	2	46	1.89	0.0323	9606.ENSP00000286648,9606.ENSP00000371230	DCK,AK3
GO:0044238	Primary metabolic process	10	7332	0.38	0.0399	9606.ENSP00000254079,9606.ENSP00000286621, 9606.ENSP00000286648,9606.ENSP00000309591, 9606.ENSP00000332116,9606.ENSP00000359719, 9606.ENSP00000366488,9606.ENSP00000367615, 9606.ENSP00000371230,9606.ENSP00000485525	PPP1R1B,ADK,DCK,PRKACA,PDE4B,PRKACB,PRKACG,APRT,AK3,ADSL
GO:0044237	Cellular metabolic process	10	7513	0.37	0.0499	9606.ENSP00000254079,9606.ENSP00000286621, 9606.ENSP00000286648,9606.ENSP00000309591, 9606.ENSP00000332116,9606.ENSP00000359719, 9606.ENSP00000366488,9606.ENSP00000367615, 9606.ENSP00000371230,9606.ENSP00000485525	PPP1R1B,ADK,DCK,PRKACA,PDE4B,PRKACB,PRKACG,APRT,AK3,ADSL

Supplementary Table 3. The enrichment process of PDE4C.

#term ID	term description	observed gene count	background gene count	strength	false discovery rate	matching proteins in your network (IDs)	matching proteins in your network (labels)
GO:0006163	Purine nucleotide metabolic process	11	329	1.77	2.32E-16	9606.ENSP00000286355,9606.ENSP00000286621,9606.ENSP00000286648,9606.ENSP00000294016,9606.ENSP00000297323,9606.ENSP00000311405,9606.ENSP00000347689,9606.ENSP00000367615,9606.ENSP00000371230,9606.ENSP00000419361,9606.ENSP00000485525	ADCY8,ADK,DCK,ADCY9,ADCY1,ADCY6,PDE4C,APRT,AK3,ADCY5,ADSL
GO:0006164	Purine nucleotide biosynthetic process	10	158	2.05	2.32E-16	9606.ENSP00000286355,9606.ENSP00000286621,9606.ENSP00000286648,9606.ENSP00000294016,9606.ENSP00000297323,9606.ENSP00000311405,9606.ENSP00000367615,9606.ENSP00000371230,9606.ENSP00000419361,9606.ENSP00000485525	ADCY8,ADK,DCK,ADCY9,ADCY1,ADCY6,APRT,AK3,ADCY5,ADSL
GO:0009152	Purine ribonucleotide biosynthetic process	9	147	2.04	7.15E-15	9606.ENSP00000286355,9606.ENSP00000286621,9606.ENSP00000294016,9606.ENSP00000297323,9606.ENSP00000311405,9606.ENSP00000367615,9606.ENSP00000371230,9606.ENSP00000419361,9606.ENSP00000485525	ADCY8,ADK,ADCY9,ADCY1,ADCY6,APRT,AK3,ADCY5,ADSL
GO:0009150	Purine ribonucleotide metabolic process	10	303	1.77	1.20E-14	9606.ENSP00000286355,9606.ENSP00000286621,9606.ENSP00000294016,9606.ENSP00000297323,9606.ENSP00000311405,9606.ENSP00000347689,9606.ENSP00000367615,9606.ENSP00000371230,9606.ENSP00000419361,9606.ENSP00000485525	ADCY8,ADK,ADCY9,ADCY1,ADCY6,PDE4C,APRT,AK3,ADCY5,ADSL
GO:0046058	cAMP metabolic process	6	22	2.69	1.68E-12	9606.ENSP00000286355,9606.ENSP00000294016,9606.ENSP00000297323,9606.ENSP00000311405,9606.ENSP00000347689,9606.ENSP00000419361	ADCY8,ADCY9,ADCY1,ADCY6,PDE4C,ADCY5
GO:1901135	Carbohydrate derivative metabolic process	11	987	1.3	3.88E-12	9606.ENSP00000286355,9606.ENSP00000286621,9606.ENSP00000286648,9606.ENSP00000294016,9606.ENSP00000297323,9606.ENSP00000311405,9606.ENSP00000347689,9606.ENSP00000367615,9606.ENSP00000371230,9606.ENSP00000419361,9606.ENSP00000485525	ADCY8,ADK,DCK,ADCY9,ADCY1,ADCY6,PDE4C,APRT,AK3,ADCY5,ADSL
GO:0034654	Nucleobase-containing compound biosynthetic process	11	995	1.29	4.02E-12	9606.ENSP00000286355,9606.ENSP00000286621,9606.ENSP00000286648,9606.ENSP00000294016,9606.ENSP00000297323,9606.ENSP00000311405,9606.ENSP00000347689,9606.ENSP00000367615,9606.ENSP00000371230,9606.ENSP00000419361,9606.ENSP00000485525	ADCY8,ADK,DCK,ADCY9,ADCY1,ADCY6,PDE4C,APRT,AK3,ADCY5,ADSL
GO:1901137	Carbohydrate derivative biosynthetic process	10	602	1.47	5.43E-12	9606.ENSP00000286355,9606.ENSP00000286621,9606.ENSP00000286648,9606.ENSP00000294016,9606.ENSP00000297323,9606.ENSP00000311405,9606.ENSP00000367615,9606.ENSP00000371230,9606.ENSP00000419361,9606.ENSP00000485525	ADCY8,ADK,DCK,ADCY9,ADCY1,ADCY6,APRT,AK3,ADCY5,ADSL
GO:0006171	cAMP biosynthetic process	5	10	2.95	2.88E-11	9606.ENSP00000286355,9606.ENSP00000294016,9606.ENSP00000297323,9606.ENSP00000311405,9606.ENSP00000419361	ADCY8,ADCY9,ADCY1,ADCY6,ADCY5
GO:0034199	Activation of protein kinase a activity	5	19	2.67	3.74E-10	9606.ENSP00000286355,9606.ENSP00000294016,9606.ENSP00000297323,9606.ENSP00000311405,9606.ENSP00000419361	ADCY8,ADCY9,ADCY1,ADCY6,ADCY5
GO:0071377	Cellular response to glucagon stimulus	5	27	2.52	1.55E-09	9606.ENSP00000286355,9606.ENSP00000294016,9606.ENSP00000297323,9606.ENSP00000311405,9606.ENSP00000419361	ADCY8,ADCY9,ADCY1,ADCY6,ADCY5
GO:0003091	Renal water homeostasis	5	37	2.38	6.33E-09	9606.ENSP00000286355,9606.ENSP00000294016,9606.ENSP00000297323,9606.ENSP00000311405,9606.ENSP00000419361	ADCY8,ADCY9,ADCY1,ADCY6,ADCY5
GO:0007190	Activation of adenylate cyclase activity	5	39	2.36	7.61E-09	9606.ENSP00000286355,9606.ENSP00000294016,9606.ENSP00000297323,9606.ENSP00000311405,9606.ENSP00000419361	ADCY8,ADCY9,ADCY1,ADCY6,ADCY5
GO:0009126	Purine nucleoside monophosphate metabolic process	5	39	2.36	7.61E-09	9606.ENSP00000286621,9606.ENSP00000286648,9606.ENSP00000367615,9606.ENSP00000371230,9606.ENSP00000485525	ADK,DCK,APRT,AK3,ADSL
GO:1904322	Cellular response to forskolin	4	11	2.81	2.36E-08	9606.ENSP00000286355,9606.ENSP00000297323,9606.ENSP00000311405,9606.ENSP00000419361	ADCY8,ADCY1,ADCY6,ADCY5
GO:0046033	AMP metabolic process	4	14	2.71	5.03E-08	9606.ENSP00000286621,9606.ENSP00000367615,9606.ENSP00000371230,9606.ENSP00000485525	ADK,APRT,AK3,ADSL
GO:0007193	Adenylate cyclase-inhibiting g protein-coupled receptor signaling pathway	5	80	2.05	1.61E-07	9606.ENSP00000286355,9606.ENSP00000294016,9606.ENSP00000297323,9606.ENSP00000311405,9606.ENSP00000419361	ADCY8,ADCY9,ADCY1,ADCY6,ADCY5
GO:0009127	Purine nucleoside monophosphate biosynthetic process	4	21	2.53	1.68E-07	9606.ENSP00000286621,9606.ENSP00000286648,9606.ENSP00000367615,9606.ENSP00000485525	ADK,DCK,APRT,ADSL
GO:0050878	Regulation of body fluid levels	7	509	1.39	5.97E-07	9606.ENSP00000286355,9606.ENSP00000294016,9606.ENSP00000297323,9606.ENSP00000311405,9606.ENSP00000367615,9606.ENSP00000371230,9606.ENSP00000419361	ADCY8,ADCY9,ADCY1,ADCY6,APRT,AK3,ADCY5
GO:0071375	Cellular response to peptide hormone stimulus	6	269	1.6	7.14E-07	9606.ENSP00000286355,9606.ENSP00000294016,9606.ENSP00000297323,9606.ENSP00000311405,9606.ENSP00000367615,9606.ENSP00000419361	ADCY8,ADCY9,ADCY1,ADCY6,APRT,ADCY5
GO:0006167	AMP biosynthetic process	3	5	3.03	1.58E-06	9606.ENSP00000286621,9606.ENSP00000367615,9606.ENSP00000485525	ADK,APRT,ADSL
GO:0007189	Adenylate cyclase-activating g protein-coupled receptor signaling pathway	5	137	1.81	1.76E-06	9606.ENSP00000286355,9606.ENSP00000294016,9606.ENSP00000297323,9606.ENSP00000311405,9606.ENSP00000419361	ADCY8,ADCY9,ADCY1,ADCY6,ADCY5
GO:0043101	Purine-containing compound salvage	3	15	2.55	1.97E-05	9606.ENSP00000286621,9606.ENSP00000286648,9606.ENSP00000367615	ADK,DCK,APRT
GO:0043174	Nucleoside salvage	3	16	2.52	2.30E-05	9606.ENSP00000286621,9606.ENSP00000286648,9606.ENSP00000367615	ADK,DCK,APRT
GO:0009116	Nucleoside metabolic process	4	104	1.84	4.68E-05	9606.ENSP00000286621,9606.ENSP00000286648,9606.ENSP00000367615,9606.ENSP00000371230	ADK,DCK,APRT,AK3
GO:0034404	Nucleobase-containing small molecule biosynthetic process	4	105	1.83	4.80E-05	9606.ENSP00000286621,9606.ENSP00000286648,9606.ENSP00000347689,9606.ENSP00000367615	ADK,DCK,PDE4C,APRT
GO:0044209	AMP salvage	2	2	3.25	0.00025	9606.ENSP00000286621,9606.ENSP00000367615	ADK,APRT
GO:0046128	Purine ribonucleoside metabolic process	3	58	1.96	0.00064	9606.ENSP00000286621,9606.ENSP00000367615,9606.ENSP00000371230	ADK,APRT,AK3
GO:0006166	Purine ribonucleoside salvage	2	5	2.85	0.00081	9606.ENSP00000286621,9606.ENSP00000367615	ADK,APRT
GO:0009153	Purine deoxyribonucleotide biosynthetic process	2	5	2.85	0.00081	9606.ENSP00000286621,9606.ENSP00000286648	ADK,DCK
GO:0007186	G protein-coupled receptor signaling pathway	6	1255	0.93	0.0029	9606.ENSP00000286355,9606.ENSP00000294016,9606.ENSP00000297323,9606.ENSP00000311405,9606.ENSP00000347689,9606.ENSP00000419361	ADCY8,ADCY9,ADCY1,ADCY6,PDE4C,ADCY5
GO:0032793	Positive regulation of creb transcription factor activity	2	19	2.27	0.0065	9606.ENSP00000286355,9606.ENSP00000297323	ADCY8,ADCY1
GO:1900273	Positive regulation of long-term synaptic potentiation	2	23	2.19	0.009	9606.ENSP00000286355,9606.ENSP00000297323	ADCY8,ADCY1
GO:0007212	Dopamine receptor signaling pathway	2	32	2.05	0.0156	9606.ENSP00000311405,9606.ENSP00000419361	ADCY6,ADCY5
GO:0007616	Long-term memory	2	34	2.02	0.0172	9606.ENSP00000286355,9606.ENSP00000297323	ADCY8,ADCY1
GO:0007610	Behavior	4	572	1.09	0.0195	9606.ENSP00000286355,9606.ENSP00000297323,9606.ENSP00000367615,9606.ENSP00000419361	ADCY8,ADCY1,APRT,ADCY5
GO:0006220	Pyrimidine nucleotide metabolic process	2	46	1.89	0.0289	9606.ENSP00000286648,9606.ENSP00000371230	DCK,AK3
GO:0061178	Regulation of insulin secretion involved in cellular response to glucose stimulus	2	57	1.8	0.0428	9606.ENSP00000286355,9606.ENSP00000419361	ADCY8,ADCY5

Supplementary Table 4. The enrichment process of PDE4D.

#term ID	term description	observed gene count	background gene count	strength	false discovery rate	matching proteins in your network (IDs)	matching proteins in your network (labels)
GO:0001993	Regulation of systemic arterial blood pressure by norepinephrine-epinephrine	2	9	2.6	0.0192	9606.ENSP00000305372,9606.ENSP00000345502	ADRB2,PDE4D
GO:0002027	Regulation of heart rate	3	99	1.73	0.0192	9606.ENSP00000309591,9606.ENSP00000345502, 9606.ENSP00000440045	PRKACA,PDE4D,ATP2A2
GO:0006163	Purine nucleotide metabolic process	4	329	1.33	0.0192	9606.ENSP00000286621,9606.ENSP00000286648, 9606.ENSP00000345502,9606.ENSP00000371230	ADK,DCK,PDE4D,AK3
GO:0006937	Regulation of muscle contraction	4	162	1.64	0.0192	9606.ENSP00000305372,9606.ENSP00000309591, 9606.ENSP00000345502,9606.ENSP00000440045	ADRB2,PRKACA,PDE4D,ATP2A2
GO:0009116	Nucleoside metabolic process	3	104	1.71	0.0192	9606.ENSP00000286621,9606.ENSP00000286648, 9606.ENSP00000371230	ADK,DCK,AK3
GO:0009126	Purine nucleoside monophosphate metabolic process	3	39	2.14	0.0192	9606.ENSP00000286621,9606.ENSP00000286648, 9606.ENSP00000371230	ADK,DCK,AK3
GO:0009153	Purine deoxyribonucleotide biosynthetic process	2	5	2.85	0.0192	9606.ENSP00000286621,9606.ENSP00000286648	ADK,DCK
GO:0019932	Second-messenger-mediated signaling	4	354	1.3	0.0192	9606.ENSP00000305372,9606.ENSP00000309591, 9606.ENSP00000345502,9606.ENSP00000440045	ADRB2,PRKACA,PDE4D,ATP2A2
GO:0032091	Negative regulation of protein binding	3	106	1.7	0.0192	9606.ENSP00000305372,9606.ENSP00000426909, 9606.ENSP00000440045	ADRB2,GNB2L1,ATP2A2
GO:0034404	Nucleobase-containing small molecule biosynthetic process	3	105	1.71	0.0192	9606.ENSP00000286621,9606.ENSP00000286648, 9606.ENSP00000345502	ADK,DCK,PDE4D
GO:0043101	Purine-containing compound salvage	2	15	2.38	0.0192	9606.ENSP00000286621,9606.ENSP00000286648	ADK,DCK
GO:0043174	Nucleoside salvage	2	16	2.35	0.0192	9606.ENSP00000286621,9606.ENSP00000286648	ADK,DCK
GO:0043393	Regulation of protein binding	4	212	1.53	0.0192	9606.ENSP00000305372,9606.ENSP00000309591, 9606.ENSP00000426909,9606.ENSP00000440045	ADRB2,PRKACA,GNB2L1,ATP2A2
GO:0044057	Regulation of system process	5	592	1.18	0.0192	9606.ENSP00000305372,9606.ENSP00000309591, 9606.ENSP00000345502,9606.ENSP00000440045, 9606.ENSP00000469689	ADRB2,PRKACA,PDE4D,ATP2A2,SHANK2
GO:0046033	AMP metabolic process	2	14	2.41	0.0192	9606.ENSP00000286621,9606.ENSP00000371230	ADK,AK3
GO:0055117	Regulation of cardiac muscle contraction	3	78	1.84	0.0192	9606.ENSP00000309591,9606.ENSP00000345502, 9606.ENSP00000440045	PRKACA,PDE4D,ATP2A2
GO:0071872	Cellular response to epinephrine stimulus	2	13	2.44	0.0192	9606.ENSP00000309591,9606.ENSP00000345502	PRKACA,PDE4D
GO:1903779	Regulation of cardiac conduction	3	68	1.89	0.0192	9606.ENSP00000309591,9606.ENSP00000345502, 9606.ENSP00000440045	PRKACA,PDE4D,ATP2A2
GO:2001257	Regulation of cation channel activity	4	177	1.6	0.0192	9606.ENSP00000305372,9606.ENSP00000309591, 9606.ENSP00000345502,9606.ENSP00000469689	ADRB2,PRKACA,PDE4D,SHANK2
GO:0009127	Purine nucleoside monophosphate biosynthetic process	2	21	2.23	0.0251	9606.ENSP00000286621,9606.ENSP00000286648	ADK,DCK
GO:0042391	Regulation of membrane potential	4	440	1.21	0.0251	9606.ENSP00000305372,9606.ENSP00000426909, 9606.ENSP00000440045,9606.ENSP00000469689	ADRB2,GNB2L1,ATP2A2,SHANK2
GO:0071880	Adenylate cyclase-activating adrenergic receptor signaling pathway	2	21	2.23	0.0251	9606.ENSP00000305372,9606.ENSP00000345502	ADRB2,PDE4D
GO:0050804	Modulation of chemical synaptic transmission	4	446	1.2	0.0253	9606.ENSP00000305372,9606.ENSP00000309591, 9606.ENSP00000440045,9606.ENSP00000469689	ADRB2,PRKACA,ATP2A2,SHANK2
GO:0006164	Purine nucleotide biosynthetic process	3	158	1.53	0.0256	9606.ENSP00000286621,9606.ENSP00000286648, 9606.ENSP00000371230	ADK,DCK,AK3
GO:0009266	Response to temperature stimulus	3	170	1.5	0.0292	9606.ENSP00000305372,9606.ENSP00000309591, 9606.ENSP00000440045	ADRB2,PRKACA,ATP2A2
GO:0010460	Positive regulation of heart rate	2	26	2.14	0.0292	9606.ENSP00000345502,9606.ENSP00000440045	PDE4D,ATP2A2
GO:0010469	Regulation of signaling receptor activity	3	176	1.48	0.0292	9606.ENSP00000305372,9606.ENSP00000345502, 9606.ENSP00000469689	ADRB2,PDE4D,SHANK2
GO:0010882	Regulation of cardiac muscle contraction by calcium ion signaling	2	26	2.14	0.0292	9606.ENSP00000309591,9606.ENSP00000440045	PRKACA,ATP2A2
GO:0016241	Regulation of macroautophagy	3	174	1.49	0.0292	9606.ENSP00000262187,9606.ENSP00000305372, 9606.ENSP00000309591	RHEB,ADRB2,PRKACA
GO:0033137	Negative regulation of peptidyl-serine phosphorylation	2	28	2.1	0.0292	9606.ENSP00000345502,9606.ENSP00000426909	PDE4D,GNB2L1
GO:0045822	Negative regulation of heart contraction	2	25	2.15	0.0292	9606.ENSP00000345502,9606.ENSP00000440045	PDE4D,ATP2A2
GO:0060314	Regulation of ryanodine-sensitive calcium-release channel activity	2	27	2.12	0.0292	9606.ENSP00000309591,9606.ENSP00000345502	PRKACA,PDE4D
GO:2000311	Regulation of ampa receptor activity	2	25	2.15	0.0292	9606.ENSP00000305372,9606.ENSP00000469689	ADRB2,SHANK2
GO:2000463	Positive regulation of excitatory postsynaptic potential	2	28	2.1	0.0292	9606.ENSP00000305372,9606.ENSP00000469689	ADRB2,SHANK2
GO:0010880	Regulation of release of sequestered calcium ion into cytosol by sarcoplasmic reticulum	2	29	2.09	0.0297	9606.ENSP00000309591,9606.ENSP00000345502	PRKACA,PDE4D
GO:0051049	Regulation of transport	6	1776	0.78	0.0362	9606.ENSP00000305372,9606.ENSP00000309591, 9606.ENSP00000345502,9606.ENSP00000426909, 9606.ENSP00000440045,9606.ENSP00000469689	ADRB2,PRKACA,PDE4D,GNB2L1,ATP2A2, SHANK2
GO:0051240	Positive regulation of multicellular organismal process	6	1770	0.78	0.0362	9606.ENSP00000262187,9606.ENSP00000305372, 9606.ENSP00000345502,9606.ENSP00000426909, 9606.ENSP00000440045,9606.ENSP00000469689	RHEB,ADRB2,PDE4D,GNB2L1,ATP2A2, SHANK2
GO:0086004	Regulation of cardiac muscle cell contraction	2	37	1.98	0.0397	9606.ENSP00000345502,9606.ENSP00000440045	PDE4D,ATP2A2
